# The Mobilisation of the Medical Profession

**Published:** 1917-02-17

**Authors:** 


### The Hospital

The Workers' Newspaper of Administrative Medicine and Institutional Life, Administration, National Insurance and Health.

No. 1602, Vol. LXI. SATURDAY, FEBRUARY 17, 1917. PRICE ONE PENNY.

### THE MOBILISATION OF THE MEDICAL PROFESSION.

For some weeks there have been busy whisperings that the Government was considering proposals for the mobilisation of the medical profession, and some of the authoritative voices of the profession have expressed sympathy with the suggestion. The report received full confirmation in the speech made by the Director of National Service when he outlined his scheme on the 7th inst. From his general appeal for volunteers for work in various industries he excluded two groups, the doctors and the ministers of religion, on the ground that both of these "seem to stand a little apart from the rest of the community in this matter, because their services are required in particular directions." The reason given for this exception is somewhat vague, but it will doubtless serve the official purpose. Translated into practical terms it means that the Army needs more doctors, that in certain districts there are doctors eligible for military service who cannot be taken for this purpose because of the wants of the civil population, and that at the same time there are other districts where the medical men are in excess. Hence the suggestion that all medical practitioners shall come under authority in order that they may be distributed in the most effective fashion to meet the wants alike of the Army and of the civil population. Every practitioner, in short, is to be ready to renounce his freedom of individual choice and to take the sphere of activity selected for him by some authority constituted for this purpose by the Government of the day. In civil practice, as in military practice, each doctor is to go where he is told however much the order conflicts with his personal convenience or interest. So far we are not told whether, as in the general scheme of National Service, the proposal is a call in the first place for volunteers with a promise of compulsion in the background. But it may be taken as certain that if other measures fail compulsion will be adopted, and by some it will be urged as more likely to secure an approximate equality of sacrifice. The broad fact is that the governing authorities are convinced of the necessity for some such scheme as the one outlined above, and they are held by their responsibility both towards the Army and the general public to endeavour to put the scheme into operation and to apply to this end the force of law if experience should show this to be necessary.

In considering this proposal it is of course necessary to remember that it is made in highly exceptional circumstances and with a view to meet what is nothing less than a national emergency. Another general consideration is that the men charged with the direction of the nation's affairs in the existing stress of events carry an exceptional burden of responsibility; there is therefore due to them a corresponding measure of authority. If the one is imposed the other must be granted. A time will come when they must give an account of their stewardship and be ready to justify their decisions. But such a time is not now. Hence, when we are plainly told that more doctors are essential to the Army it is little to the purpose for individuals to rush to the newspapers with complaints of defects in the existing organisation or with tales of military doctors who have little or nothing to do. Let these failures be ventilated by all means, but their value in the last resort must be fixed by the Army chiefs, and when, in spite of the critics, these authorities repeat that they need more medical help, this help, both the public and the profession will agree, must be forthcoming. It is merely an extension of this argument to say that the wants of the Army can only be safely met by an authoritative redistribution of the profession according to the needs of the civil population as these needs exist when military necessities have been satisfied. Opposition on principle to all conscription and compulsion may be understood even by the most enthusiastic advocates of these methods, but it is difficult to find a cloak of respectability for the claim that while others should suffer such pressure we ourselves should be exempt. To fight and to care for the fighter are national demands abundantly sanctioned by the nation's struggle for existence. To act in home duties as substitutes for men who have undertaken these tasks stands on the same plane, and if compulsion can be justified in the one case it can equally be justified in the other. Even those who urged that the medical profession should only come under this claim when the general population was similarly treated can no longer oppose the proposal, for their condition is satisfied. For our own part we could not sympathise with this demand, as the medical profession has very special obligations in wartime, and we cannot imagine the profession refusing to perform these on the ground that those with more limited opportunities were allowed freedom of choice. However, in principle, there is now no room for contention except for those—they must be few in number—who are able to say that from the outset they have opposed all methods of compulsion applied to the individual for purposes of war service.

There are, in our judgment, two conditions on which, while accepting the principle of mobilisation, the profession should insist. The first is that the administration of the scheme shall be in the hands of a professional body, for none other would really be competent to meet the difficult and delicate situations which will undoubtedly arise. The second condition is that the scheme shall be in operation only for the duration of the war, and that so soon after this as possible there shall be a re-establishment of existing conditions. We profess no enthusiasm for mobilisation. To us it can be defended only as an emergency measure necessary for the safety of our armies and people. Under the very best administration it will, we have no doubt, inflict hardship and inconvenience on individual doctors, and will produce results justifying dissatisfaction and annoyance on the part of patients. When the risk of such evils has disappeared the new discipline will have lost its justification. It is the more necessary to say this because there is a school of political thought which rejoices at the prospect of getting the medical profession into the bondage of official chains, and hopes that once applied these chains will be permanent. For ourselves we have a profound distrust of a creed which teaches that wisdom dwells only in committees or boards, and that the individual cannot be trusted with the choice of his own fate.

